# Health deficits in community dwelling adults aged 40 to 75 years

**DOI:** 10.1186/s12877-019-1152-9

**Published:** 2019-05-27

**Authors:** Susan Gordon, Michael Kidd, Anthony Maeder, Nicky Baker, Tania Marin, Karen Grimmer

**Affiliations:** 1 College of Nursing and Health Sciences, Flinders University, Adelaide, South Australia 5042 Australia; 2 Professor and Chair, Department of Family & Community Medicine, University of Toronto, Toronto, Canada; 3 Honorary Professor of Global Primary Care, Southgate Institute for Health, Society and Equity, Flinders University, Adelaide, South Australia Australia; 4 Digital Health Research Centre, College of Nursing and Health Sciences, Flinders University, Adelaide, South Australia Australia

**Keywords:** Healthy ageing, Screening assessments, Normal values, Health status

## Abstract

**Background:**

Middle and older years are associated with age related health deficits but how early this begins and progresses is poorly understood. Better understanding is needed to address early decline and support healthier ageing outcomes.

**Methods:**

Seemingly healthy, community dwelling adults aged 40 to 75 years were recruited via local council and business networks. They completed online surveys about sleep quality, distress and physical activity, and two hours of objective testing of physiologic and anthropometric measures, mobility, cognition, grip strength, foot sensation, dexterity and functional hearing. Analysis compared outcomes for age, gender, and age and gender groups with population norms for 21 health assessments. The total number of non-compliant tests for each participant was calculated by summing the number of non-compliant tests, and the frequency of these scores across the sample was reported. Gender and age effects were tested using ANOVA models. Combined age and gender categories were used for subsequent logistic regression modelling, with females aged 40–49 years being the default comparator.

**Results:**

Of 561 participants (67% female; mean age 60 years (SD 10.3)), everyone had at least one deficit and median deficits was 5 (IQR 2). More than 50% of participants did not meet anthropometric and exercise norms, while 30 to 40% had reduced functional hearing and cognition. Overall, men performed worse and deficits increased with age particularly for physical activity, audiology, mobility, anthropometry, oximetry and foot sensation. Heart rate, body temperature and dyspnoea were the only variables where compliance was within 95% of expected values. Multiple areas of functional decline were found in people aged in their 40s and 50s.

**Conclusions:**

The health deficits identified are mostly mutable hence identification and interventions to address the multi-system functional decline in people as young as 40 has the capacity to ensure healthier ageing.

## Background

Healthy ageing is defined as the ongoing process of optimising physical, mental, social and emotional function that enables wellbeing as individuals age [1]. Functional decline is believed to occur when deterioration in physical, mental, social or emotional performance significantly impacts on an individual’s capacity to live independently [2]. Such changes are commonly reported as correlates of the ageing process [3].

However, there is no clear point-in-time when age-related changes to body systems performance commence. Little is known about when, or what physiological and functional changes can be detected in generally healthy adults living and ageing independently in the community. Age-related decline in function can occur independently of body systems changes resulting from disease [3, 4]. This is why early age-related changes in individuals living independently in the community are often not detected by healthcare professionals, who generally only consult people when they are unwell [3]. Moreover, there is no agreed way to comprehensively assess early functional decline because ageing involves multiple body systems, and age-related changes manifest differently in different individuals [4]. It is not surprising therefore, that there has been little focus on middle-aged (40–59 years) and young-old people (50–75 years) to determine when and how, early physiological and functional changes related to ageing are detectable, and whether these changes can be reversed.

Healthy ageing is a global priority. The 69th World Health Organization (WHO) Assembly adopted a global strategy and action plan on ageing and health [5] and proposed a decade of healthy ageing (2020 to 2030) to highlight the global importance of ensuring healthy older age. WHO called on all partners to participate in research and innovation to foster healthy ageing, “… including developing: (i) *evidence-based tools to assess and support clinical, community and population-based efforts to enhance intrinsic capacity and functional ability; and (ii) cost-effective interventions to enhance functional ability of people with impaired intrinsic capacity”* [5]. Any opportunity to prevent, reverse or halt poor body systems performance can only have positive outcomes for the individual, the community, the health system and the economy more broadly [6–8].

One way of screening for poor body systems performance is to compare individual performance with expected population norms, on an understanding that population norms reflect an expected, acceptable range of performance in a healthy (or normal) population. Glasser first coined the term ‘*norm-referenced test’* [9]. These are tests with population norms which provide values that indicate when individuals are not operating within expected population ranges for healthy body systems. Population norms are usually developed from repeated population sampling and testing, generally using data from large randomized controlled trials or observational studies. They can also be derived from data syntheses from systematic reviews of such studies. [10].

This paper reports on findings from a large population-based study of seemingly healthy, community-dwelling Australians aged 40 to 75 years in one Australian capital city, to measure body systems performance and compare with established population norms. Participants whose performance failed to meet expected population norms were hypothesized to show signs of early functional decline and hence their possible entry onto the trajectory of early ageing [1, 2].

## Method

### Ethics, consent and permissions

Provided by the Southern Adelaide Local Health Network (South Australia, Australia) (391.16). This paper conforms to the principles embodied in the Declaration of Helsinki. Return of online surveys implied consent. All participants provided signed consent prior to objective assessment, which included use of data for publication.

### Study design

Cross-sectional observation study.

### Population norms for measures of aging

A systematic literature review [11] layered with expert panel input identified health screening assessments for attributes of ageing, with 21 having published and validated population norms [12] (see Table 2). The systematic review identified six broad health domains of early functional decline. These comprised medical status (biological systems); performance capacity (physical, cognition and mental constructs); participation (environment, function and motivation constructs); as well as demographics, anthropometry and relationships with health providers [11]. The expert panel identified gaps in body performance assessment for dental health, vision, reaction time and upper limb dexterity, appetite and nutrition. Instruments to assess these constructs were identified from additional targeted literature searches. The final set of assessments is reported elsewhere [12].

### Additional data items

To investigate potential correlates of non-compliance with population norms, or expected normal values, age and gender were included.

### Recruitment

Adults aged 40–75 years living independently in the community were purposively recruited through extensive partnerships with local government and a national bank. The aim of recruitment was to attract people who would not normally come to medical providers’ attention for ill health, or public health officers’ attention for comprehensive health screening.

### Aims

This paper reports:

1. how well community-dwellers aged 40–75 years in one Australian capital city complied within 95% of expected healthy population norms of body systems performance and.
2. age and gender influences on non-compliance with population norms.

### Data collection

Data was collected between January and June 2017. Participants completed an online or hard copy self-reported survey prior to attending an objective testing session (subjective and objective assessments are denoted in Table 1). Before objective measures participants were screened for potential risk of adverse events using physiological measures. Where one or more of these variables was outside the expected norms, participants were counselled about seeking medical help and subsequent screening assessments were modified.

**Table 1:** Gender differences and mean age for number of deficits

Deficits	%Males	%Females	mean age (SD)
1	0	0.3	60 (0)
2	0.6	2.2	55.7 (11.6)
3	4.7	7.7	56.0 (9.9)
4	7	15.3	59.9 (11.9)
5	15.2	20.8	61.6 (9.9)
6	12.9	20.8	60.7 (9.9)
7	18.1	15.6	59.3 (9.8)
8	18.1	10.1	60.0 (9.9)
9	11.7	3.6	60.0 (10.1)
10	7.6	1.9	62.3 (6.7)
11	1.7	0.8	60.8 (4.5)
12	1.7	0.6	55.0 (12.6)
13	0.6	0	49 (0)

*SD* standard deviation

### Data management

Data were recorded in Microsoft Access™. Responses to the online surveys, and the objective data were linked using the unique participant code. Data were analysed using IBM SPSS for Windows and SAS Version 9.1. Missing data were examined for extent and pattern.

The primary study outcome of compliance with each of the population thresholds was reported in binary form (Yes, No) and a summary score of non-compliance was calculated, with a possible maximum of 21, based on a score of one for each assessment where a participant did not meet expected norms. The other explanatory variables were age groups (reported as 40–49; 50–59; 60–69, 70 years and over) and gender.

### Data analysis

Non-compliance with each healthy population norm were described as percentages. Differences in frequencies of compliance (or not) for each assessment were calculated using chi square models based on an hypothesis of no difference between frequencies (significant differences set at *p* < 0.05). The total number of non-compliant tests for each participant was calculated by summing the number of non-compliant tests, and the frequency of these scores across the sample was reported. Gender and age effects were tested using ANOVA models.

To address the second aim, logistic regression models were constructed. Age groups and gender categories were treated firstly as independent variables because there was clear evidence from our literature search that males and females aged differently (11). Using odds ratios to identify risk differences in a population, by necessity, takes an abstract approach which requires a default reference standard. The default comparator choice while operational was in this instance based on the youngest age group being more likely to meet or be close to expected values than the older age groups and would therefore identify differences between age groups. Hence for age analysis, the youngest age group (40–49 years) was designated as the default comparator for age group associations. Age and gender associations were then considered using the eight independent categories (female with each age group and male with each age group) for subsequent logistic regression modelling, with females aged 40–49 years being the default comparator for operational purposes. For chi square, ANOVA and regression models, significance was identified at *p* < 0.05. For logistic regression models, significance was identified when two tailed 95% Confidence Intervals (95%CI) around an odds ratios (OR) did not encompass the value 1.

## Results

### Demographics

Of the 561 participants 547 provided data on all 21 population norms. Females represented 67% of the sample, and sample mean age was 60 years (Standard Deviation (SD) 10.3) Despite a significantly greater number of females than males (*p* < 0.05) in the 40–49 and 75+ age groups the percentages of males and females in the sample did not differ from those reported by the Australian Bureau of Statistics (ABS) for metropolitan Adelaide (2016). Thus, this sample was likely to provide a reasonable estimate of population characteristics.

### Compliance with healthy population norms

**Fig. 1:**
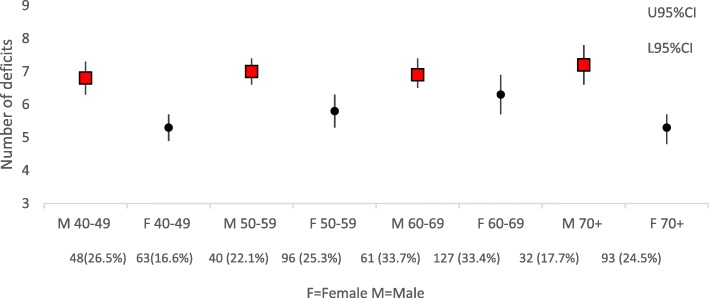
Mean number of deficits by age and gender. F=Female, M = Male>

No participant complied with all healthy population norms. The maximum number of non-compliant tests (deficits) was 13, with the most commonly occurring number being five (reported by 19.0% sample). There were significant independent gender and age effects on the total number of deficits, but there were no gender or age-gender interaction effects. The frequency of per-person total deficits is reported in Fig. 1, and the percentage of males and females, and mean age (SD) for each number of deficits is reported in Table 1.

Table 2 reports each of the health measures, the source of the population norm values, the number of participants who did not meet the expected norm, the independent effects of age group and gender, and the combined effects of gender and age group (default comparison category women aged 40–49 years, OR = 1) on each measure. Five percent error was applied regarding absolute compliance with each population norm (seeking 95% compliance or better). Heart rate, body temperature and dyspnoea were the only variables where compliance was within 95% of expected values. Significant independent gender differences were found for compliance with healthy population norms for women (poorer findings compared with men of physical activity, mobility, waist circumference) and for men (poorer findings compared with women for hearing, body mass index (BMI), waist-hip ratio and foot sensation. Increasing age was significantly and independently associated with increasing hearing problems, decreased mobility, high BMI, poor oximetry, lower waist-hip ratios and reduced foot sensation. Differences between gender-age groups were found for lung function, hearing, blood pressure, mobility, waist-hip ratio, foot sensation and waist circumference.

**Table 2:** Health screening measurements, source of population norm data, compliance with population norms, and age and gender effects on non-compliance. An OR less than 1 indicates that males are more likely than females to have the outcome and an OR greater than 1 indicates that females are more likely to have the outcome than males

Measure	Non-compliant *n* (%)	Independent age group effect	Independent gender effect	Age and gender subgroups
		(OR 95% CI)	(OR 95% CI)	(OR 95% CI)
		Comparator 40–49 y	Comparator Female	Comparator Female 40-49y
Overweight or obese*	338 (61.1)	40-49y OR = 1	0.7 (0.5–1.0)	
BMI 25–29.9 (overweight), 30+ (obese) [13]		*50-59y 1.7 (1.1–2.9)*		
		*60-69y 1.8 (1.2–2.9)*		
		*70 + y 2.2 (1.3–3.9)*		
Waist-hip ratio	298 (53.1)	40-49y OR = 1	0.2 (0.1–0.3)	Compared with F 40-49y:
		*50-59y 0.5 (0.3–0.7)*		M40-49y 0.2 (0.07–0.4)
High risk: > 0.90 for males; > 0.85 for females [13]		60-69y 1.0 (0.6–1.7)		M50-59y 0.3 (0.1–0.5)
		70 + y 1.1 (0.7–3.9)		M60-69y 0.6 (0.3–0.3)
Physical activity* (see Footnote for description)	293 (52.9)	No significant age effects	1.5 (1.1–2.1)	
Blood Glucose 3.0–7.7 mmol/L [16]	216 (38.5)	No significant age effects	No significant gender effects	
Audiology*	188 (33.9)	40-49y OR = 1	0.6 (0.4–0.9)	Compared with F40-49y:
7 or 8 = pass [17]		*50-59y 2.5 (1.2–5.0)*		F 50-59y 3.1 (1.2–8.1)
		*60-69y 7.6 (3.9–14.6)*		F 60-69y 6.0 (2.4–15.1)
		*70 + y 6.7 (3.4–13.3)*		F 70 + y 7.3 (2.9–18.6)
				M 60-69y 20.0 (7.3–54.5)
				M 70 + y 8.4 (2.8–24.9)
Waist circumference*	185 (33.4)	No significant age effects	2.1 (1.4–3.2)	Compared with F 40-49y:
High risk: females: ≥ 88 cm				M 40-49y 0.04 (0.01–0.1)
males: ≥102 cm or more [13]				M 50-59y 0.1 (0.05–0.3)
				M 60-69y 0.14 (0.1–0.3)
				M70 + y 0.1 (0.05–0.2)
Cognition and memory*	179 (32.3)	No significant age effects	No significant gender effects	
GPCog <=8 [18]				
Blood pressure*	164 (29.6)	40-49y OR = 1	No significant gender effects	Compared with F 40-49y:
90-140 mmHg systolic, 60-90 mmHg diastolic [16]		50-59y 1.3 (0.7–2.3)		F 70 + y 2.5 (1.2–5.3)
		60-69y 1.5 (0.9–2.5)		M 60-69y 2.7 (1.2–6.1)
		*70 + y 1.9 (1.1–3.5)*		
Mobility*	140 (25.3)	40-49y OR = 1	1.4 (1.0–2.2)	Compared with F 40-49y:
Six Minute Walk Test (6MWT)		50-59y 1.3 (0.8–3.1)		F 70 + y 4.1 (1.8–8.9)
6MWDm = 518.853 + (1.25 x height in cm) – (2.816 x age in years) – (39.07 x sex men = 0; women = 1) [19, 20]		*60-69y 2.3 (1.2–4.3)*		
		*70 + y 3.8 (1.9–7.4)*		
Foot sensation*	123 (22.6)	40-49y OR = 1	0.6 (0.4–0.9)	Compared with F 40-49y:
Mono-filament testing All 20 correct responses (10 each foot) [21		*50-59y 2.2 (1.0–4.5)*		F 60-69y 3.6 (1.3–9.9)
		*60-69y 2.9 (1.5–5.9)*		F 70 + y 4.2 (1.5–11.6)
		*70 + y 4.1 (2.0–8.5)*		M 50-59y 4.0 (1.6–12.8)
				M 60-69y 4.8 (1.6–14.1)
				M 70 + y 10.2 (3.2–32.4)
Perceived exertion*	115 (20.5)	No significant age effects	No significant gender effects	
Borg Exertion Scale				
scale 6–20, at-risk values> 13 [22]				
Sleep quality*	113 (20.4)	No significant age effects	No significant gender effects	
Pittsburgh Sleep Quality Index)(PSQI) ≥ 8 [23, 24]				
Lung capacity*	82 (14.8)	No significant age effects	No significant gender effects	Compared with F 40-49y)
FEV1, FVC Normative tables for age and gender see [25]				M 40-49y 3.9 (1.3–11.9)
				M 50-59y 3.9 (1.2–12.3)
Dexterity*	82 (14.4)	No significant age effects	No significant gender effects	
The Purdue Dexterity Test norms see [26]				
Grip strength* (dominant hand) Hand held dynamometer	74 (13.4)	No significant age effects	No significant gender effects	
At-risk is in lowest 20th% of gender-age distribution [27, 28]				
Respiratory rate*	17 (13.1)	No significant age effects	No significant gender effects	
12–20 breaths/min [16]				
Oximetry*	36 (6.5)	40-49y OR = 1	No significant gender effects	
< 96% [16]		50-59y 6.1 (0.73–50.3)		
		60-69y *1.8 (1.5–90.4)*		
		*70 + y 9.7 (1.2–77.3)*		
K10 Psychological Distress*	32 (5.8)	No significant age effects	No significant gender effects	
≥ 13 [29]				
Dyspnoea	8 (1.4)	No significant age effects	No significant gender effects	
Borg Dyspnoea Scale At-risk values > 4 [22]				
Temperature	3 (0.5)	No significant age effects	No significant gender effects	
35.5–37.5 degrees C [16]				
Heart rate	4 (0.4)	No significant age effects	No significant gender effects	
60–100 beats per min [16]				

Key: Significant differences between expected compliance and percentage of non-compliant scores (*p* < 0.05) identified by* after each measure; significant age group effects identified by italics: *CI* confidence interval, *F* female, *M* male, *OR* odds ratio, *y* years

Footnote: Active Australia Survey 18–65 year olds at risk: < 150 to 300 min (2 ½ to 5 h) moderate intensity physical activity, or 75 to 150 min (1 ¼ to 2 ½ hours) of vigorous intensity physical activity, or combination of both, each week. 65+ years at-risk: < 30 min moderate intensity physical activity on most days [14, 15]

The three healthy body performance thresholds that were least well complied with, across the sample, were recommended BMI (61.1% participants being overweight or obese), recommended waist-hip ratio (53.1% exceeding this) and recommended physical activity thresholds (52.9% not meeting them). Of importance to this research is that neither age nor gender was associated with compliance with healthy population thresholds for psychological distress, lung function, sleep quality, cognition, grip strength, respiratory or heart rate, temperature, blood glucose levels, dexterity, perceived exertion or dyspnoea.

## Discussion

This paper reports new and important information on compliance with 21 published healthy population screening thresholds, in a large sample of community-dwelling Australians, aged 40–75 years in one Australian capital city. The significant influence of increasing age on compromised hearing, high blood pressure, high BMI, low oximetry, large waist circumference, foot sensation and mobility is reasonably explained by aging body systems [3, 30]. However, this study highlights the need for ongoing investigations into early onset of potentially age-related body performance changes, as it found that poor body systems performance (usually associated with older age), can be detected in community-dwelling people as young as 40 years. Non-compliance with at least one healthy population threshold was found in 97.3% of our sample (reflecting men and women of any age), for psychological distress, lung function, sleep quality, cognition, grip strength, respiratory rate, heart rate, temperature, physical exertion, dyspnoea and dexterity. It is essential to better understand why these comparatively young people, who should be experiencing good health and physical fitness, fail to meet one or more of the expected healthy body performance thresholds.

The body performance measures that we used reflect expected, healthy population norms, and are all potentially mutable. Thus, targeted interventions could be provided to redress declining function in one or more body systems, detected in our sample. Health promotion programs, conducted in the community or in workplaces, could readily target the most common poorly-performing body systems (for instance those with non-compliance rates greater than 30%), such as high BMI and large waist circumference, cognition, poor physical activity and poor hearing. In doing this, other body performance measures may also be improved by default (such as walking distance and speed, blood pressure, lung function and sleep quality (all scoring 20–30% non-compliance in our study)). Such interventions may well reverse or delay early ageing signs, and increase propensity for healthier aging in middle-aged people (40–59 years). This study allowed us to identify those tests where deficits are most likely to be found with respect to both age and gender. This information can be used to design future health screening programs that are shorter and targeted, and hence more efficient and affordable.

That approximately one in five participants reported poor foot sensation is concerning, particularly as decreased sensation is associated with decreased blood flow, peripheral neuropathy and diabetes [29]. The threshold of healthy foot sensation was to correctly identify 20 monofilament applications to the sole of the foot [21]. Thus, participants scoring 19/20 were considered not to meet the population norm, which may be too stringent for population testing. Whilst we believe that the test was delivered reliably, the testing procedure could be reconsidered for possible application error, and participant response variability on repeated testing. Moreover, an allowable number of non-correct responses may need to be determined for future population screening, to allow for possible misunderstanding and/or misclassification of participant responses. Despite this, people recording less than expected healthy foot sensation should be referred for further testing, to eliminate (or address) sinister underlying causes.

The volunteer nature of participants potentially introduces biases, including expecting a benefit, and presenting with undeclared disease [31]. Whilst we are confident that our sample was generally reflective of 2016 local age-gender demographics, it potentially over-represents the population in one of two ways: attracting healthy people (who wanted validation of their ‘health’ state) or attracting unhealthy people wanting further information and understanding of their health concerns [31]. The partner organisations in this study provided support for recruitment and did not, or could not, allow access to comprehensive registers of information on people’s date of birth. Thus, the recruitment strategy (using emails, media and posters) was the best within available resources, and cannot account for people who did not hear about the study (but would have participated had they known), and people who chose not to participate in the study (and for what reason). Furthermore, information was not collected on why people volunteered.

## Conclusion

This study adds comprehensive information about functional decline in multiple body systems in seemingly healthy, community dwelling, middle-aged and young-older-aged adults aged 40–75 years. Based on published population norms, this study demonstrates multiple areas of functional decline occurring in people who would not expected to demonstrate them. Most areas of identified functional decline are however, amenable to change with known interventions.

There is no public health screening agenda in Australia to comprehensively identify early functional deficits. Poor compliance with population health norms in this group would not be recognised until a significant health event. Education and interventions to address these early health deficits would support healthier ageing.

## Abbreviations

ABS: Australian bureau of statistics
ANOVA: Analysis of variance
BMI: Body mass index
CI: Confidence Interval
F: Female
IBM: International business machines corporation
M: Male
OR: Odds ratio
SAS: Statistical analysis system
SD: Standard deviation
SPSS: Statistical package for the social sciences
WHO: World health organization
Y: Years
